# The RNA-Binding Protein HuR in Digestive System Tumors

**DOI:** 10.1155/2020/9656051

**Published:** 2020-07-24

**Authors:** Xiaoqing Song, Xin Shi, Wenjuan Li, Fa Zhang, Zhonglin Cai

**Affiliations:** ^1^Department of Pathology, College of Basic Medical Sciences, China Medical University, Shenyang, Liaoning 110000, China; ^2^Department of General Surgery, Luzhou People's Hospital, Luzhou, Sichuan 646000, China; ^3^Department of Respiratory and Critical Care Medicine, Henan Provincial People's Hospital; People's Hospital of Zhengzhou University; People's Hospital of Henan University, Zhengzhou, Henan 45003, China; ^4^Department of Urology, Lanzhou General Hospital of Lanzhou Military Command, Lanzhou, Gansu 730050, China

## Abstract

Human antigen R (HuR) is a member of the Hu family of RNA-binding proteins. This molecule, which was first described in tumors nearly two decades ago, has recently received much attention in tumor-related research because it regulates the expression of many tumor-associated molecules through posttranscriptional regulatory mechanisms, thereby affecting biological characteristics. It is suggested that HuR might be a novel therapeutic target and a marker for therapeutic response and prognostic assessment. Increasing evidence supports that HuR also plays critical roles in the development, therapy, and prognosis of digestive system tumors. Herein, we review the relationships between HuR and digestive system tumors, demonstrating the importance of HuR in digestive system tumor diagnosis.

## 1. Introduction

Human antigen R (HuR) is an RNA-binding protein (RBP) belonging to the human embryonic lethal abnormal visual (ELAV) protein family that was originally identified as a specific antigen in patients with paraneoplastic neurological symptoms [1, 2]. Unlike other ELAV family members (i.e., HuB, HuC, and HuD) that are specifically expressed in neurons, HuR is widely expressed in a variety of tumors and is mainly present in the cytoplasm [3]. With the deepening of research, HuR has been shown to be related to not only the development, angiogenesis, apoptosis, invasion, and metastasis of various malignant tumors but also tumor chemotherapy, radiotherapy resistance, and patient prognosis [4–6]. It is a novel tumor treatment target and a marker for treatment response and prognostic evaluation.

## 2. HuR Background

### 2.1. Structure and Function of HuR

**Figure 1:**
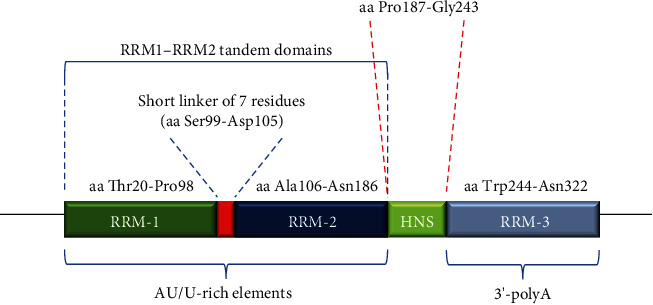
RNA recognition sequences of HuR consist of RRM-1, RRM-2, and RRM-3. The RRM-1-RRM-2 tandem domain is composed of RRM-1, a short linker of 7 residues, and RRM-2 [9]. RRM-1 and RRM-2 can bind to AU/U-rich elements, and RRM-3 can bind to the polyadenylation tail of rapidly degraded mRNA. Abbreviations: RRM: RNA recognition sequence; aa: amino acid; HNS: HuR nucleocytoplasmic shuttling sequence.

RBPs that mediate gene expression and posttranscriptional regulatory mechanisms play important roles in affecting the biological characteristics of tumors. Among them, HuR has been shown to be an important posttranscriptional regulator as an RBP [7]. It is encoded by the HuR gene located on chromosome 19, 19p13.2, and it has a molecular weight of approximately 32 kD and is overexpressed in almost all malignancies [7]. In normal resting cells, HuR is mainly located in the nucleus, and under the action of various stimulating factors, HuR binds to its target mRNA to form an HuR-mRNA complex, which is transported to the cytoplasm to exert its function of stabilizing the target mRNA and regulating protein translation [8]. These mRNAs are characterized by adenosine/uridine- (AU-) or uridine- (U-) rich elements (AU/U-rich elements, also known as AREs), which are recognized by and bound to HuR through three classical RNA recognition sequences (RRMs): RRM-1 and RRM-2, which bind to AU/U-rich elements, and RRM-3, which binds to the polyadenylation tail of rapidly degraded mRNA [8]. Furthermore, it has been identified that the target mRNA sequence capable of binding to the RRM on HuR is a U-rich sequence of approximately 17-20 nucleotides in length that is mainly located in the 3′ untranslated region (3′-UTR) of the target mRNA (Figure 1) [9].

Studies have shown that HuR is involved in the regulation of the expression of many genes and that changes in its protein levels or subcellular localization are associated with many human diseases, such as pathological inflammation, atherosclerosis, and ischemia [10, 11]. In addition, the target mRNAs of HuR are transcripts encoding oncogenic factors, including oncogenes, growth factors, and antiapoptotic factors [12, 13]. Therefore, the high expression of HuR in tumor cells compared with normal cells suggests that it plays a key role in tumor progression, and cytoplasmic HuR accumulation in malignant tumors (including pancreatic cancer, lung cancer, gallbladder cancer, and urothelial cancer) is related to poor prognosis [14–17]. In summary, an increasing number of studies have confirmed that HuR plays a role as an oncogene and plays a crucial role in tumor progression.

### 2.2. Regulatory Mechanism of HuR Expression

Although HuR-mediated posttranscriptional regulation plays a key role in the expression of many transcripts, the regulation of the functions and expression of HuR is complex and remains to be clarified. First, HuR modulators, including Smad, TTP, RNP C1, Mdm2, pp32, Hsf1, and NO, bind to the GC-rich 5′-UTR of the HuR mRNA and increase the expression of HuR mRNA [18–20]. In addition, miRNAs, including miR-519, miR-125a, miR-9, miR-16, miR-29a, and miR-200c, can inhibit the translation and expression of HuR [21, 22]. In addition to regulating the expression of HuR, the process that shuttles HuR from the nucleus to the cytoplasm, where it plays its corresponding role, is regulated by several endogenous or exogenous stimuli, such as insulin or DNA damage [23]. Many signaling pathways, including members of the mitogen-activated protein kinase (MAPK) or protein kinase C (PKC) family, have been implicated to be involved in the regulation of intracellular HuR localization [24]. Finally, some proteins, such as pp32 and leucine-rich acidic protein (APRIL), appear to bind to specific HuR regions and alter their ability to translocate to the cytoplasm [25, 26]. In summary, the regulation of HuR function is associated with changes at the protein level and the regulation of nuclear shuttling. However, the elucidation of detailed regulatory mechanisms still requires further studies.

## 3. The Role of HuR in Affecting the Biological Characteristics of Digestive System Tumors

HuR is significantly increased in digestive system tumors, including liver cancer, pancreatic cancer, colorectal cancer, gastric cancer, and esophageal cancer, and it is mainly located in the cytoplasm. HuR affects the biological characteristics of digestive system tumors by regulating the expression of a variety of tumor-related molecules that are involved in tumor development and tumor cell apoptosis, invasion, migration, and proliferation. Interestingly, cumulative studies have shown that HuR promotes cell senescence, which is closely related to tumors, by increasing reactive oxygen species (ROS) by enhancing the mitochondrial localization of the telomeric protein TIN2 and increasing Foxp3 expression, activating oncogene-induced senescence via the Ca^++^-CaMKK*β*-AMPK*α*2-HuR pathway, etc. [27–29]. Therefore, studying the functional properties of HuR plays a crucial role in the future prevention and treatment of tumors.

### 3.1. HuR and Liver Cancer

The expression of HuR in liver cancer tissues is significantly higher than that in normal liver tissues [30], and HuR is concentrated in the cytoplasm; moreover, the HuR staining score (0-1 or 2-3) in early- and late-stage liver cancer tissues has a positive association with disease stage (0, early (I/II) or late (III/IV)) [31]. This study also showed that cytoplasmic HuR is expressed more highly in hepatocellular carcinoma than in normal liver tissue and that high HuR staining scores are associated with decreased survival in patients with hepatocellular carcinoma [31]. Basic studies have shown that HuR overexpression promotes the conversion between methionine adenosyltransferase 1A (MAT1A) and methionine adenosyltransferase 2A (MAT2A) [32]. It is well known that MAT2A is mainly expressed in the fetal liver and is gradually replaced by MAT1A as the fetus grows [33]. The expression of MAT2A is upregulated during liver regeneration and the dedifferentiation of liver cancer cells [33]. Thus, HuR plays a key role in liver dedifferentiation and in the development and progression of hepatocellular carcinoma through the posttranscriptional regulation of MAT1A and MAT2A mRNAs [32]. In addition to promoting the growth of liver cancer, HuR is also closely related to the invasive ability of liver cancer. A study of the mechanism of hepatitis B virus- (HBV-) related hepatocellular carcinoma indicated that HBV-encoded X protein (HBx) induces HuR expression and mediates the increased stability of epidermal growth factor receptor (EGFR) mRNA [34]. Regarding EGFR protein expression, EGFR overexpression promotes the migration of hepatocellular carcinoma expressing HBx. HuR also binds to the 3′-UTR of matrix metalloproteinase-9 (MMP-9) mRNA to increase the stability of MMP-9 mRNA, promote the expression of MMP-9, and increase the invasive ability of hepatocellular carcinoma Hep3B cells [35]. In liver cancer, HuR promotes tumor progression and is regulated by other molecular mechanisms. Liu et al. showed that miR-16 not only directly inhibits COX-2 expression in hepatoma cells but also indirectly inhibits COX-2 by downregulating HuR expression [36]. The downregulated expression of COX-2 increases the apoptosis of liver cancer cells and reduces cell proliferation [36]. In addition, the mechanism by which lincRNA-UFC1 promotes HCC growth also involves HuR, and the mechanism by which lincRNA-UFC1 directly interacts with HuR to regulate the expression level of *β*-catenin in HCC cells plays a role in promoting HCC [37].

### 3.2. HuR and Pancreatic Cancer

Chronic inflammation is one of the predisposing factors of tumors. The overexpression of HuR in the mouse pancreas leads to a pancreatic fiber inflammatory response and other pathological features of chronic pancreatitis, which is closely related to the occurrence of pancreatic cancer [38]. The KrasG12D gene mutation is one of the initiation events of cancer, and the overexpression of HuR in combination with the KrasG12D mutation in the pancreas resulted in a 3.4-fold increase in the incidence of pancreatic ductal adenocarcinoma (PDAC) compared with the KrasG12D mutation alone [39]. These data indicate that HuR is closely related to the susceptibility to pancreatic cancer and has a potential role as a carcinogenic factor in pancreatic tumors. Recently, psychological stress has been associated with increased adrenaline, which has been shown to be a risk factor for the development of pancreatic cancer [40]. Adrenalin increases the migration of pancreatic cancer cells by activating the TGF*β* pathway, increasing the risk of distant metastasis by inducing the nuclear mass translocation of HuR [40]. The increase in the neurotransmitter adrenaline leads to poor survival in patients with pancreatic cancer [40]. Similarly, Jimbo et al., using small interfering RNAs to silence HuR expression, also demonstrated a significant reduction in tumor growth characteristics, such as the proliferation, migration, and invasion of pancreatic cancer cells [41]. HuR not only affects pancreatic cancer development and cell proliferation, migration, and invasion but also may be a biomolecular marker for evaluating treatment response and prognosis. Oba et al. studied 141 patients with PDAC who underwent surgery, and 61 of these patients received gemcitabine. The results showed that the disease-free survival (DFS) rate of patients treated with gemcitabine was significantly higher in patients with HuR overexpression than in those in the control group and that high HuR expression had a positive association with a good reaction to gemcitabine treatment [42]. In addition, Tatarian et al. performed HuR immunolabeling in 379 PDA patients and divided the patients into a high-expression group and a low-expression group. The results showed that there was no difference between gemcitabine (GEM) and 5-fluorouracil (5-FU) treatments in the low-expression group, but in the high-expression group, the median DFS was significantly elevated in 5-FU-treated patients and was higher than that in GEM-treated patients (*P* = 0.04) [43]. These findings suggest that HuR has the potential to serve as a biomarker for predicting prognosis and for the assessment of therapeutic response or as a promising target for multidrug combination therapy. However, detailed mechanisms and large-scale clinical trials are needed to confirm these findings.

### 3.3. HuR and Colorectal Cancer

As early as 2000, researchers showed that HuR can target cyclins A and B1 to promote the proliferation of colorectal cancer RKO cells [44]. The treatment of CRC cells with the HuR small-molecule inhibitor MS-444 resulted in increased tumor cell growth inhibition and apoptosis [45]. In addition, MS-444 inhibited CRC xenograft tumor growth by enhancing apoptosis and reducing angiogenesis after the intraperitoneal administration of MS-444 [45]. Interestingly, the cytoplasmic expression of HuR was also significantly associated with increased COX-2 expression and a higher tumor stage. HuR binds to the COX-2 mRNA 3′-UTR to promote COX-2 translational expression [46], while MS-444 inhibits the nuclear mass transfer of HuR in cells to further reduce the expression of COX-2, exerting a tumor-suppressing effect [45].

In addition to regulating downstream tumor-promoting factors, HuR is also regulated by a variety of other factors. As a tumor-promoting factor, miR-155-5p was found to be a functional target of the HuR mRNA 3′-UTR, and the targeted inhibition of miR-155-5p reduced HuR expression and the migration of colon cancer HT-29 cells [47]. The phosphorylation of protein molecules is the main form of action of many drug molecules. HuR is affected by the phosphorylation of protein kinase C*δ*, which changes its function and expression. Compared with the colonic epithelial cell line CCD 841, the phosphorylation of nuclear HuR in colon cancer DLD-1 cells is more obvious [48]. In addition, a significant increase in the phosphorylation of HuR was also found in tissue specimens of colon cancer. Functionally, phosphorylated HuR was able to cause a significant increase in the migration and proliferation of CCD 841 cells [48]. HuR is also targeted by tumor suppressors to inhibit tumor growth. The tumor suppressor miR-22 has been found to target HuR to inhibit CRC cell proliferation and migration and reduce the growth of colorectal xenograft tumors [49].

The expression of HuR and the cytoplasmic abundance of HuR increase with the degree of tumor malignancy, which is related to the survival rate of colorectal cancer patients [50]. Yoo et al. analyzed the relationship between HuR expression and survival in specimens from 560 colorectal cancer patients, and the results showed that high levels of HuR expression were associated with late-stage disease characteristics and were a predictor for poor survival in patients with colorectal cancer [51]. In summary, HuR is closely related to the biological characteristics of colorectal cancer, and the in-depth study of its role in tumorigenesis is of great significance for the prevention and treatment of tumors.

### 3.4. HuR and Gastric Cancer

Compared with normal gastric tissue, HuR is significantly increased in gastric cancer tissues, especially in advanced tumors [52]. The high expression level of HuR in the nucleus is correlated with the invasion depth, TNM stage, and tumor size, while the extent of HuR accumulation in the cytoplasm is associated with lower patient survival. Moreover, a number of studies have also shown that cytoplasmic HuR expression is associated with decreased survival in gastric cancer patients [53–55]. The above finding suggests that HuR overexpression and subcellular localization are inversely related to clinicopathological features and patient prognosis [56]. At the cellular level, the overexpression of HuR increases the proliferation of tumor cells, activates the G1/S transition of the cell cycle, and promotes DNA synthesis and cell growth [52, 57]. In contrast, silencing its expression by siRNA reduced the proliferation of tumor cells and inhibited the response to apoptotic stimuli [52]. Wang et al. showed that HuR promotes the proliferation and migration of gastric cancer cells by upregulating high-mobility group protein 1 (HMGB1) [56], but the detailed mechanism remains to be further studied. Interestingly, it has been shown that HuR is related to the potential effects of oncogenic viral infections, such as inflammation and oncogenicity [58–60]. Epstein-Barr virus (EBV) is well known to be associated with lymphomas, gastric cancer, nasopharyngeal carcinoma, etc. HuR promoted EBV-mediated cellular transformation by binding to the EBV stable intronic sequence (sis) [61]. Therefore, virus-induced oncogenicity is closely related to the deregulation of HuR. The above results indicate that HuR is closely related to gastric cancer and is expected to be a target in future research.

### 3.5. HuR and Esophageal Cancer

Clinical pathological studies of esophageal squamous cell carcinoma (ESCC) showed that HuR is overexpressed in the cytoplasm of tumor cells and that cytoplasmic HuR expression is positively correlated with lymph node metastasis and the depth and stage of tumor invasion [61]. Multivariate analysis showed that cytoplasmic HuR expression is an independent prognostic factor for esophageal cancer and that its expression is associated with low survival rates in esophageal cancer [61]. Recently, a similar study also indicated that HuR protein and mRNA levels were higher in esophageal cancer tissues than in adjacent tissues [62]. The downregulation of HuR significantly inhibited cell proliferation and migration, and the mechanism may be related to the expression of HuR regulating matrix metalloproteinase 2 (MMP2), MMP9, and vimentin [62]. In addition, the overexpression of HuR leads to the increased stability of survivin mRNA and protein expression in esophageal cancer cells, which in turn regulates tumor cell apoptosis and promotes tumor growth [63]. Therefore, HuR is also related to the biological characteristics of esophageal cancer. However, the relevant regulatory mechanisms still need to be confirmed in further studies.

## 4. The Role of HuR in the Treatment of Digestive System Tumors

HuR affects the biological characteristics of tumors; however, an increasing number of studies have shown that HuR is associated with tumor chemotherapy, radiotherapy, and endocrine therapy tolerance. This suggests that HuR can be used as a novel adjuvant therapeutic target to further improve the therapeutic effect of tumor treatments.

### 4.1. HuR and Chemotherapy

Chronic hypoxia is strongly associated with cytotoxic chemotherapy and radiochemotherapy tolerance, a phenomenon known as hypoxia-induced chemotherapy resistance. Pancreatic ductal adenocarcinoma exhibits high levels of hypoxia characterized by hypoxic conditions (pO_2_) and reduced intracellular perfusion of O_2_ [64]. Studies have shown that in pancreatic cancer, HuR binds to the 3′-untranslated region of PIM1 mRNA, a key regulatory molecule involved in hypoxia-induced chemoresistance, and upregulates its expression [64]. The HuR small-molecule inhibitor MS-444 can affect HuR-mediated PIM1 overexpression by inhibiting HuR homodimerization and its cytoplasmic translocation, thereby enhancing the sensitivity of pancreatic cancer PDA cells to oxaliplatin/5-fluorouracil under physiological hypoxic conditions [64]. HuR not only protects pancreatic cancer cells from hypoxia but also protects tumors from chemotherapy tolerance in low-nutrition conditions. Due to nutrient deficiencies and chemotherapy leading to a surge in reactive oxygen species (ROS), the adaptive mechanisms required for pancreatic cancer cells to maintain oxidative stress in the microenvironment contribute to chemotherapy resistance [65]. HuR mediates pancreatic cancer chemotherapy resistance by regulating the expression of isocitrate dehydrogenase 1 (IDH1) under low-nutrient conditions [65]. Furthermore, in pancreatic cancer, HuR is associated with the chemosensitivity of pancreatic cancer by targeting deoxycytidine kinase (dCK), a key enzyme that activates gemcitabine [66, 67]. HuR may also mediate pancreatic cancer gemcitabine resistance by targeting COX-2 [68, 69]. The knockdown of HuR significantly increases the sensitivity of colon cancer cells to apoptosis induced by the chemotherapy drugs doxorubicin and paclitaxel [70]. The mechanism by which HuR mediates the tolerance of digestive cell tumors may be related to the regulation of the expression of ABCG2, galectin-3, *β*-catenin, cyclin D1, Bcl-2, P-gp, MRP1, and MRP2, which mediates the resistance of colorectal cancer to chemotherapy drugs [5, 71, 72]. Therefore, the inhibition of HuR expression is an important strategy to reverse the resistance of gastrointestinal tumors. Therapeutic strategies targeting HuR are expected to be safer and more effective treatments. However, detailed and in-depth preclinical studies and large-scale clinical trials still need to be carried out.

### 4.2. HuR and Radiotherapy

Radiotherapy is an important means of tumor treatment. After tumor radiotherapy, some tumor cells resistant to radiotherapy survive and become the root of tumor recurrence [73]. Therefore, exploring the mechanisms of radiotherapy tolerance and finding a strategy to enhance the sensitivity of radiotherapy have become current research priorities. Recently, studies on the relationship between HuR and radiation therapy have indicated that the expression of caspase-2 in colorectal cancer cells is significantly increased after silencing HuR and that the colorectal cancer cells DLD-1 and HCT-15 are more sensitive to radiation-induced apoptosis [74]. In addition, a decrease in HuR significantly increases the number of radiation-induced *γ*H2AX/53BP1-positive foci, suggesting an increase in DNA damage, and it is believed that HuR is involved in the mRNA expression of caspase-2, which is involved in the repair of DNA damage and interferes with the expression of HuR [74, 75]. This leads to a decrease in the expression of caspase-2, which leads to DNA instability in tumor cells, mediates DNA damage accumulation in tumor cells, and causes cell death [74]. In a study on liver cancer and HuR, HuR was found to reduce the sensitivity of liver cancer cells to radiotherapy by promoting the mRNA expression of mitochondrial transcription factor A (TFAM), which is associated with decreased radiosensitivity, thus affecting the effect of radiotherapy [6]. The above results indicate that HuR plays an important role in tumor radiotherapy tolerance. Targeting HuR is expected to be a potential strategy for increasing the sensitivity of radiotherapy.

## 5. Conclusions

The HuR protein has the ability to translocate from the nucleus to the cytoplasm under various stimulating factors, thereby stabilizing target mRNA. Posttranscriptional modifications appear to control the abundance and localization of HuR and its binding affinity to target mRNA. Therefore, HuR upstream regulatory molecules, background expression levels, nuclear-cytoplasmic translocation, etc. may be important therapeutic strategies for reducing HuR-mediated tumor growth. With the deepening of tumor-related research, HuR is becoming an attractive target for therapeutic digestive system tumor treatments. However, detailed questions about HuR in the digestive system remain to be explored.
